# Misdiagnosis of Bell's palsy: Case series and literature review

**DOI:** 10.1002/ccr3.2832

**Published:** 2020-04-16

**Authors:** Colin Bacorn, Nancy Su Teng Fong, Lily Koo Lin

**Affiliations:** ^1^ Department of Ophthalmology and Vision Science University of California Davis Health Sacramento CA USA; ^2^ University of California Davis School of Medicine University of California Davis Sacramento CA USA

**Keywords:** Bell's palsy, cranial neuropathy, facial paralysis, infection, tumor

## Abstract

Although Bell's palsy is a common etiology for isolated facial paralysis, it is important clinicians perform a complete neurologic examination to avoid misdiagnosis. Multiple cranial neuropathy is often caused by tumor or infection.

## Graphical abstract

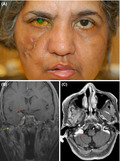

## 1 INTRODUCTION

Facial paralysis is a common neurologic symptom with potential for diagnostic error. We present 2 patients misdiagnosed with Bell's palsy and reviewed reported cases. Several exhibited multiple cranial neuropathies with more ominous pathology. This report illustrates the importance of thorough neurologic examination and the need for precise language in clinical practice.

Acute facial paralysis is a common neurologic condition whose underlying etiology may have significant morbidity and mortality. With an annual incidence of approximately 15‐30 in 100 000 Bell's palsy is the most common reported cause of acute facial paralysis accounting for 60%‐80% of cases.^1^, ^2^, ^3^, ^4^ While Bell's palsy is a benign condition with recovery in 85% of patients, its high prevalence may contribute to physicians' failure to recognize more insidious masquerades of this benign and idiopathic condition.^5^ Meticulous examination of the cranial nerves and careful consideration of the case history is essential to identify patients likely to have a more dangerous cause of their facial palsy. In addition to historical features, such as a chronic or subacute onset of symptoms and prior malignancy, involvement of additional cranial nerves should lead providers to view a diagnosis of Bell's palsy with suspicion. Multiple cranial neuropathy is under recognized to the patients' detriment as the underlying cause is often a potentially life‐threatening tumor or infection.^6^, ^7^ Usage of the term “Bell's Palsy” indiscriminately for all patients with facial paralysis may contribute to cognitive biases and discourage practitioners from pursuing further workup. The use of more precise language in both clinical documentation and the published literature can help minimize this concern. This report presents two recent cases where patients with multiple cranial neuropathy were misidentified as isolated facial nerve palsy followed by a review of the current literature.

## 2 METHODS

This case series and literature review describe two patients who were diagnosed with Bell's palsy and ultimately found to have multiple cranial neuropathies, including their clinical presentation, workup, treatment, and outcomes. An extensive review of the literature published before October 2019 was conducted by searching the PubMed database for reports of patients diagnosed with Bell's palsy that went on to have an underlying identifiable cause for their facial palsy. The search terms “bell's palsy,” “bell's palsy misdiagnosis,” “bell's palsy mimic,” “facial palsy misdiagnosis” were used to identify potentially relevant reports. Non‐English language publications were excluded as were any publication for which the full text was not available for review. Candidate publications were then screened for relevance based on title and abstract and relevant papers were reviewed in full with attention to the documentation of the patients’ evaluation on presentation and final diagnoses.

## 3 CASE PRESENTATIONS

### 3.1 Case 1

A 50‐year‐old female with no ocular history and a medical history of diabetes mellitus presented to the oculoplastic service for management of right eye lagophthalmos due to Bell's palsy. The patient reported that 4 months prior to presentation her right face became swollen and painful following a tooth extraction. This facial swelling was attributed to a dental abscess, and she went on to require intensive care unit (ICU) level care at an outside hospital for cellulitis in the setting of diabetic ketoacidosis. Her swelling resolved with antibiotic therapy, and she followed with her primary medical doctor for the next four months (a total of eight office visits with no documentation of a cranial nerve examination) for persistent complaint of right‐sided facial pain and weakness. Concurrently, she was followed by an ophthalmologist (three visits) for difficulty closing her right eye. Lagophthalmos and corneal exposure with inferior corneal scarring were noted but neither the patient's visual acuity nor the function of cranial nerves other than the facial nerve were documented at these ophthalmologic evaluations. Both providers documented concern for “Bell's Palsy,” and the patient was treated with oral corticosteroids. After 4 months, her medical doctor recommended neuroimaging (magnetic resonance imaging [MRI]) but she was referred to oculoplastics for further management of her persistent lagophthalmos prior to obtaining this study.

**Figure 1:**
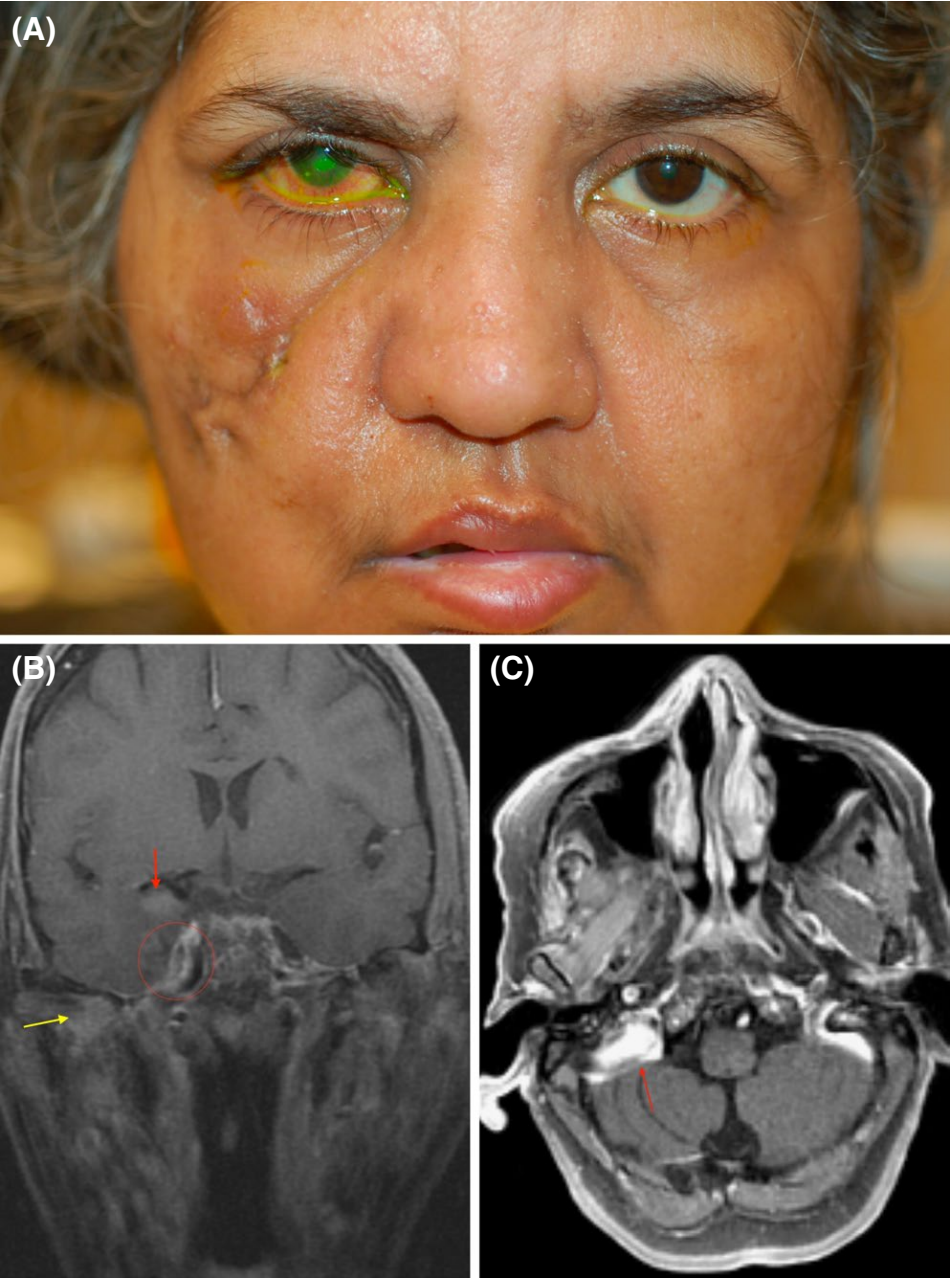
A, External photograph demonstrating right facial paralysis with facial scarring from prior cellulitis, right keratopathy with fluorescein staining. B, Coronal T1‐weighted magnetic resonance image with fat‐saturation and gadolinium (FSGD) demonstrating pterygopalatine fossa involvement (yellow arrow) with dural and perineural enhancement (red circle), and temporal lobe (red arrow). C, MR Axial T1 FSGD showing enhancement of the cerebellopontine fossa (red arrow)

On presentation, she had no light perception visual acuity in the right eye. Her examination was also significant for House‐Brackmann grade 4 palsy of the right facial nerve and loss of sensation in the V1 distribution of the right trigeminal nerve.^8^ (Figure 1A) The severity of keratopathy limited posterior examination, but the right optic nerve appeared pallid. Further workup was performed, and a dedicated MRI of the orbit with and without gadolinium contrast demonstrated optic nerve atrophy, abnormal enhancement along the trigeminal nerve with extension to Meckel's cave, and edema of the right temporal lobe concerning for perineural spread and cerebral parenchymal involvement of an invasive malignancy (Figure 1B‐C) Subsequent transnasal endoscopic biopsy of the pterygopalatine fossa demonstrated fungal elements which were identified as Mucorales species, and she was diagnosed with chronic mucormycosis of the right muscles of mastication and skull base. She was treated with amphotericin and micafungin and has remained symptomatically and radiographically stable in follow up for over two years.

### 3.2 Case 2

A 59‐year‐old female with a medical history of chronic hepatitis C was referred for management of a right nonhealing neurotrophic ulcer due to Bell's palsy. One‐year prior, she reported experiencing “lightning bolt” pains across the right side of her face. She was referred to a neurologist who did not note any abnormality of facial sensation or motor function at that time; a brain MRI was obtained and was unremarkable (this study was not dedicated to the fifth nerve, and no comment was made on structures inferior to the skull base in this report). She was diagnosed with trigeminal neuralgia and treated with carbamazepine. Subsequently, she noted a decrease in visual acuity and was evaluated by optometry where a serological workup for temporal arteritis was ordered and returned negative. She went on to develop right hearing loss and noticed right temporal wasting. She developed a right‐sided facial paralysis and returned to her neurologist who now documented right fifth and seventh nerve palsies. As a result, she underwent a lumbar puncture with normal protein and cell counts as well as negative results for HSV, West Nile, and tuberculosis. At this time, her ophthalmologist independently documented right facial weakness, decreased facial sensation, and neurotrophic keratitis consistent with fifth and seventh nerve palsies. The patient was told her symptoms were a result of Bell's palsy. No repeat neuroimaging was obtained after the onset of her facial weakness. She was treated with pressure patching of the affected eye but, due to a lack of resolution, was ultimately referred for management of the neurotrophic ulcer.

**Figure 2:**
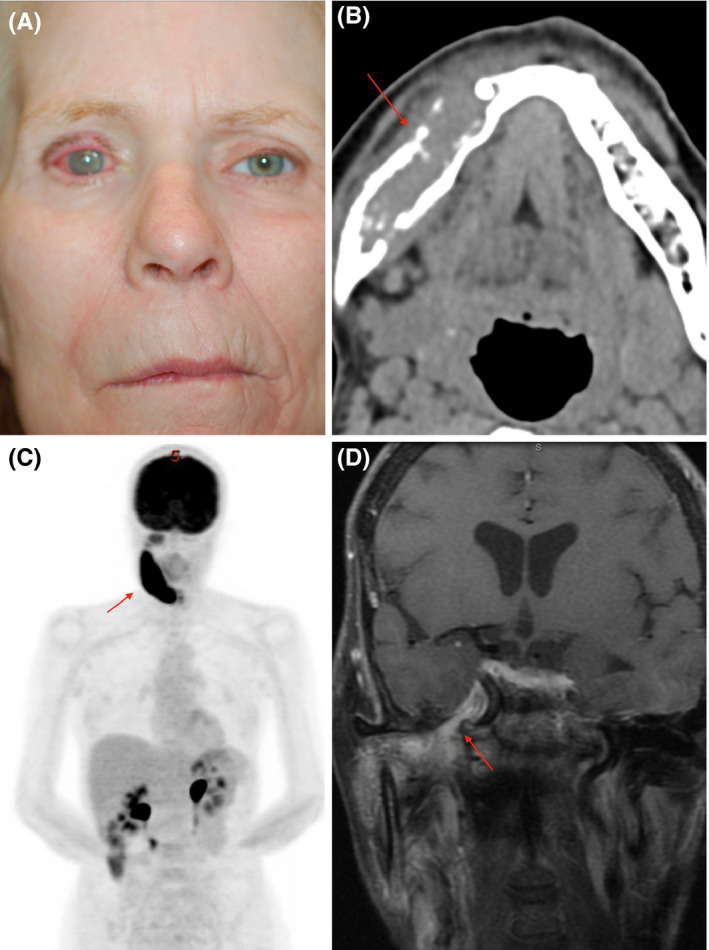
A, External photograph showing right keratopathy, facial nerve paralysis, and temporal wasting (B). Axial computed tomography (CT) demonstrating an osseous lesion of the right mandible (red arrow). C, Fluorodeoxyglucose (FDG)‐positron emission tomography (PET) with FDG uptake of the right mandible (red arrow). D, MR coronal T1 FSGD with enhancement of the trigeminal nerve (red arrow)

On presentation to the oculoplastic service, visual acuity was count fingers and she demonstrated 6 mm of lagophthalmos, a poor Bell's reflex, and a large central corneal epithelial defect (Figure 2A) Incomplete facial and trigeminal nerve palsies were diagnosed on the basis of lagophthalmos and reduced corneal sensation, and additional workup with neuroimaging was urgently obtained to determine the underlying etiology. Maxillofacial computed tomography with contrast demonstrated a lytic right mandibular lesion at the level of the ramus (Figure 2B). Fluorodeoxyglucose (FDG)‐positron emission tomography (PET) scan revealed uptake in the right mandible (Figure 2C). A biopsy of this lesion demonstrated spindle cell sarcoma with perineural spread along the trigeminal nerve. Following her diagnosis, she developed a right abducens nerve palsy concerning for further extension, and MRI of the orbit, face, and neck demonstrated involvement of the 5th and 7th nerve with intracranial spread and enhancement of the right pons and middle cerebral peduncle (Figure 2D). Her malignancy was deemed inoperable due to its extent, and she went on to receive palliative chemoradiation and then expired one and a half years after presentation.

## 4 LITERATURE REVIEW

3015 unique publications were identified and screened as described in the Methods section. Eighty‐seven records related to the misdiagnosis of Bell's palsy and were further reviewed. Twenty‐four of these papers reported cases of patients misdiagnosed with Bell's palsy (25 unique individuals) with sufficient detail to document that a dedicated examination of the patient's cranial nerves had been performed at presentation. The patient information for the twenty‐five patients reported is summarized in Table 1. While all patients exhibited motor signs/symptoms attributable to the facial nerve, other symptoms related to the nerve's sensory function or nonspecific symptoms were not uncommon at presentation. (Table 2) The original authors' identified several common factors contributing to a misdiagnosis of Bell's palsy in their discussions. The most frequently suggested factor was the presence of a particularly rare disease or presentation in six cases (Lee,^9^ Narayanan,^10^ Ivanković,^11^ Hiraumi,^12^ Torrealba‐Acosta,^13^ Torres^14^). This was followed by false negative or misinterpreted neuroimaging studies in four cases (Freije,^15^ Agarwal,^16^ Özkale,^17^ Hiraumi^12^) and a failure to recognize physical examination findings/historical features in an additional four cases (May,^18^ Blevins,^19^ Brach,^20^ Orhan^21^).

**Table 1:** Reported Bell's palsy mimics

Case	Year	Author	Age	Sex	Laterality	Nerve	Diagnosis
1	1972	May^18^	58	M	R	7	Basal cell carcinoma
2	1992	Blevins^19^	59	F	L	7	Pleomorphic adenoma
3	1996	Freije^15^	71	M	R	7	Parotid adenocarcinoma
4	1999	Brach^20^	36	M	R	5, 7	Adenoid cystic carcinoma
5	2006	Reid^39^	6	F	R	7, 12	Rhabdomyosarcoma
6	2006	Rafii^40^	44	F	R,L	7	Neurosarcoidosis
7	2006	Jain^41^	34	F	L	7	Neurosarcoidosis
8	2007	Lee^9^	28	F	L	7	Inflammatory pseudotumor
9	2008	Narayanan^10^	35	F	B	7	Guillain‐Barre syndrome
10	2008	Orhan^21^	45	F	L	7	Parotid Abscess
11	2009	Kaushal^42^	19	M	R	7	Aseptic Meningitis
12	2011	Agarwal^16^	47	M	L	7	Dorsal pontine infarct
13	2011	Ivankovic^11^	35	F	R	7	Multiple Sclerosis
14	2012	Hadano^43^	85	F	R	7	Septic emboli
15	2013	Greenberg^44^	52	M	R,L	3, 5, 7, 11	Neuroborreliosis
16	2013	Ozkale^17^	<1	F	R	7	Germ cell tumor
17	2014	Hiraumi^12^	76	M	L	7	Primary CNS lymphoma
18	2014	Hiraumi^12^	60	F	R	7	Small cell lung cancer
19	2016	Villeneuve^45^	79	F	L	7	Breast cancer metastasis
20	2017	Zheng^46^	53	M	L	7	Ramsay‐Hunt syndrome
21	2017	Nishiguchi^47^	54	M	L	7	Guillain‐Barre syndrome
22	2017	Mok^48^	73	M	R	7	Invasive aspergillus
23	2018	Karadan^49^	46	M	L	7	Pontine hemorrhage
24	2018	Torrealab‐Acosta^13^	67	M	L	7	Waldenstrom's macroglobulinemia
25	2019	Torres^14^	21	M	R,L	5, 6, 7	Polyneuritis cranialis

**Table 2:** Presenting symptoms and signs

Symptom and sign	Cases (#)
Facial paralysis, Asymmetry, or Lagophthalmos	25
Pain, Numbness, or Paresthesia	8
Nausea, vomiting, or vertigo	3
Drooling, dysphagia, speech change, or taste change	3
Epiphora	1
Hyperacusis	1
Other	8

## 5 DISCUSSION

The appropriate triage of patients with acute facial paralysis is a common challenge for physicians in a variety of specialties and care settings. Bell's palsy is the single most common etiology for this presentation but other diagnoses must be excluded.^22^ In the emergency setting, a recent review identified a misdiagnosis rate of <1% but previous literature has suggested rates as high as 20% with 5% of lower motor facial paralysis being due to tumor.^23^, ^24^ Li et al found that one‐third of the patients in a cohort of facial nerve schwannomas had been initially misdiagnosed as Bell's palsy.^25^ It is also important to recognize patients with true Bell's palsy in order to avoid unnecessary and potentially harmful treatment such as the administration of thrombolytics.^26^, ^27^, ^28^ The American Academy of Neurology (AAN) and American Academy of Otolaryngology Head and Neck Surgery Foundation (AAO‐HNSF) have published guidelines regarding the appropriate treatment of Bell's palsy.^29^, ^30^ Both societies recommend administration of oral steroids (prednisone 50‐60 mg daily) and, less strongly, antiviral medications (acyclovir or valacyclovir) to patient's with new (defined as <72 hours onset by AAO‐HNSF) Bell's palsy. The AAO‐HNSF recommends reassessment or referral to a specialist in cases with new or worsening symptoms following diagnosis, ocular symptoms, or incomplete recovery after 3 months. While the AAO‐HNSF specifically recommends against obtaining neuroimaging in acute Bell's palsy, it is recommended to pursue imaging in atypical cases (recurrent ipsilateral paralysis, incomplete facial paralysis, multiple cranial neuropathy, or duration greater than 3 months).

Factors leading to diagnostic error may be multiple and difficult to specifically identify. Review of the cases presented in this manuscript suggests that failure to perform, or incorrect interpretation of, the physical examination contributed to diagnostic error in both cases. This factor, as well as the factors suggested by the authors identified by our literature review, has been noted as contributors to diagnostic error by other reports as well. While advanced imaging studies may be appropriate in select cases, they are not infallible and up to 12.5% of malignant causes of facial nerve palsy have been missed by MRI.^31^ As in these reported cases, a decline in physical examination skill has been implicated in a large proportion of diagnostic error.^32^ Clinicians may be aided applying traditional teachings, such as that central causes of facial paralysis “spare the forehead,” but these guidelines are not absolute and careful examination of the cranial nerves is indicated for any patient presenting with facial paralysis.^33^ These concerns are not limited to patients presenting with facial paralysis, and the Institute of Medicine has previously highlighted diagnostic error as a significant contributor to patient morbidity and mortality as well as the highest proportion of paid malpractice claims.^34^

Multiple cranial neuropathy, like Bell's palsy, may be idiopathic but is more commonly caused by tumor and infection.^6^, ^7^, ^35^ Secondary cases most commonly involved the third, fifth, sixth and seventh nerves, a pattern also observed in a pooled cohort from two small studies of idiopathic cranial polyneuropathy.^35^, ^36^ While idiopathic cases have a better prognosis, with complete recovery in the majority of cases, secondary causes generally have a much poorer prognosis given the close anatomy of the skull and face.

**Figure 3:**
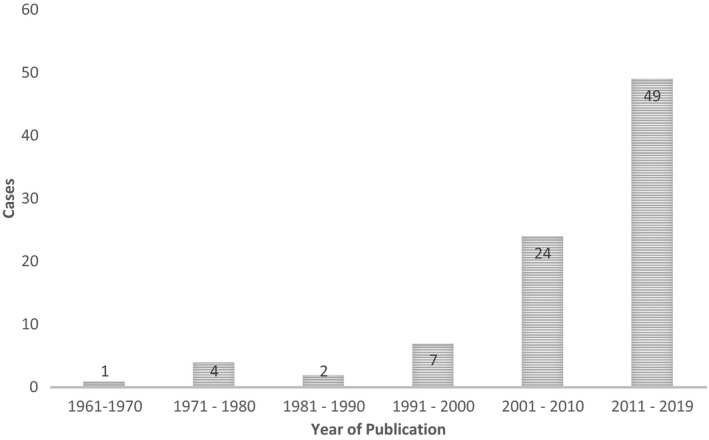
Distribution of reported cases of Bell's palsy misdiagnosis in the past 58 years

The term Bell's palsy must be used with care in communication with other providers and in the literature as it implies a thorough and negative examination to exclude other entities. In the absence of documentation of this examination, referral centers must view this diagnosis with caution. A recent report from a facial nerve referral center may support this notion by reporting significantly lower proportions of patients with Bell's palsy than historical case series.^37^ Fuller et al have suggested that the incautious use of the term Bell's palsy can lead to inappropriate patient care by leading physicians to ignore alternate diagnoses.^38^ In our own literature review, fewer than 30% of the screened papers concerning patients with presumed Bell's palsy documented complete examination of the cranial nerves yet six of these 27 patients demonstrated involvement of multiple cranial nerves, highlighting the potentially missed diagnostic opportunity. Further dialogue regarding diagnostic error is needed. Awareness of these issues in the medical community may be rising as evidenced by the increase in publications regarding Bell's palsy mimics over the past 50 years (Figure 3). Facial paralysis is common with a broad differential diagnosis but, despite the increasing availability of medical imaging and serologic investigations, the astute physician may be best served by the consistent and thorough physical examination of all such patients.

## CONFLICT OF INTEREST

None declared.

## AUTHOR CONTRIBUTIONS

CB: designed the work; acquired, analyzed, and interpreted the data; and drafted the work. NF: acquired and analyzed the data and drafted the work. LKL: conceived the work and critically revised and approved the final published version.

## Data Availability

Data sharing is not applicable to this article as no new data were created or analyzed in this study.
